# Resource allocation in decision support frameworks

**DOI:** 10.1186/s12962-018-0128-5

**Published:** 2018-11-09

**Authors:** Charles Phelps, Guruprasad Madhavan

**Affiliations:** 1 University of Rochester, Rochester, NY USA; 2 National Academies of Science, Engineering and Medicine, Washington, DC USA

**Keywords:** Multi-criteria decision analysis (MCDA), Cost effectiveness analysis (CEA), Cost–benefit analysis (CBA), Budget constraints, Quality-adjusted life years (QALYs), Policy development, Investment planning, Portfolio analysis

## Abstract

**Background:**

Cost–benefit and cost-effectiveness analysis place limits on the dimensions of value that the models can incorporate. Cost–benefit analysis requires monetization of all measures of value (including life), a task sometimes deemed either difficult to accomplish or even repugnant. Cost-effectiveness analyses include health care gains in natural units (e.g., quality-adjusted life years or QALYs) rather than purely monetizing them (e.g., in dollars) and offers an efficiency perspective based on the ratio of cost per QALYs or similar health measures. These two methods use different rules for investment. Cost–benefit analysis says to invest whenever benefits exceed costs. Cost-effectiveness analysis says to invest if the intervention has a cost per QALY that meets—or is below—a designated cutoff value.

**Methods:**

Multi-criteria frameworks expand decision analyses by considering value tradeoffs from decision makers, and then producing a synthetic measure that summarizes the performance of investment options. This evaluation is done across all chosen dimensions of value, based on the weights provided by the decision makers, but this flexibility comes at a cost. To date, no approach is widely accepted to suggest how much to invest (how to determine a budget constraint) using multi-attribute models. Moreover, there is no agreed-upon method to measure willingness to pay for incremental multi-attribute value improvements. Our paper proposes a way forward.

**Results:**

Based on existing dollar estimates of willingness to pay for QALYs, our concept creates a comparable cutoff for multi-criteria value measures. Our proposed method expands the acceptable cost per QALYs in proportion to how much of the total measure is accounted for by the QALY component. Agreed-upon values for cost per QALY are thus extrapolated to account for extra value created by non-QALY attributes of each intervention.

**Conclusion:**

Using our proposed methods, the cost per QALY cutoff can serve as a benchmark toward creating a resource allocation cutoff in multi-criteria frameworks.

## Background

Methods to evaluate and allocate societal investments have evolved over time. Cost–benefit analysis grew from the work of French engineer-economist Jules Dupuit in 1844 [1], later formalized by economist Alfred Marshall [2]. The US government began the specific requirement of cost–benefit analyses for water navigation projects in 1936, further codified in the 1939 Flood Control Act, which embodied into law an operative investment decision rule: Invest when “the benefits to whomever they accrue [be] in excess of the estimated costs” [3]. This illuminates an important limitation of the cost–benefit analysis: it cannot consider the distribution of those benefits and costs, yet issues of distribution and equity are the center of many public policy debates.

Planners and analysts of health care have been reluctant to fully embrace the concept of cost–benefit analysis, since it requires an explicit statement by the analyst of the value of a human life or life-year. Over time, cost-effectiveness emerged as an appealing criterion, wherein the analyst can evaluate the incremental costs and health benefits of various medical interventions and then report their ratio. Decision makers then set the cutoff value for approval of investments.

The proof that this approach flowed directly from a single person’s lifetime utility maximization program came only in 1997 [4]. Until that point, the intuitive appeal of cost-effectiveness was all that supported its legitimacy. The use of cost-effectiveness significantly expanded during the latter part of the twentieth century, most notably within the British National Health Service which—through its National Institute for Health and Care Excellence—evaluates medical interventions using a cutoff value currently set around £30,000 per quality adjusted life-years (QALYs) [5]. The World Health Organization recommends the use of modified cost-effectiveness analysis to evaluate health care interventions: using disability-adjusted life years (DALYs), it recommends a cutoff value of one to three times per-capita gross domestic product to guide resource allocation [6].

Once a cutoff value for an acceptable investment has been made, cost-effectiveness analysis and cost–benefit analysis are virtually equivalent, with the difference being the choice for the value measure, be it lives, life-years, QALYs or DALYs [7]. That said, how does one establish the cost-effectiveness cut-off? A general discussion of this issue appears in the most recent cost-effectiveness “handbook” [8]. A complication arises when a health system announces one cost-effectiveness threshold (e.g., $100,000 per QALY) but establishes a budget that is insufficient to fund all technologies passing the established threshold. This creates an affordability conundrum.

This issue has recently even entered the British court system with a lawsuit over prescription drugs facing a conflict between cost-effectiveness thresholds and affordability [9]. If the budget is too tight to fund all approved technologies, then that implies a more stringent threshold in actual use. In all that follows, we intend to use the constraint that binds more tightly (typically the budget). Embedded in a tight budget the “shadow price” for QALYs—the price that really matters. So if the official cutoff is $100,000 per QALY and the budget only would fund activities with cost-effectiveness ratios of $80,000 per QALY, then we would intend that the $80,000 value be used.

Both cost–benefit and cost-effectiveness approaches share a common defect: they are narrow, and cannot include practical critical factors such as the distribution of benefits and costs, broader impact of societal programs, public values and perceptions, and related matters [10]. The importance of these distributional issues have been considered [8, 11–13]. One model measures the distribution of health benefits and costs across various population subgroups [14] but does not provide a mechanism to synthesize this information into a unified value measure.

More comprehensive decision support systems such as multi-criteria decision analysis can include these issues such as equity and social distribution directly and transparently [15–17]. Multi-criteria approaches have demonstrated their value, especially when decision makers have various—and often competing—priorities [18].

Multi-criteria models differ in an important way from standard economic analyses. Economists typically *estimate* the structure of people’s preferences from observed choices they make using the formal tools of utility maximization and demand theory. These “revealed preferences,” as economists call it, are inferred from actual choices. Multi-criteria decision analysis does something completely different: it *elicits* the preferences of the decision makers and the trade-offs that they are willing to make. Subsequently, most of these approaches use a simplified method for approximating the total value of any portfolio, often using linear approximation. These “attributes” might include considerations relating to finances, health gains, social justice, or patient preferences to avoid certain side effects of treatments some of which could (in theory) be included in formal cost-effectiveness models (with relevant data) but often are omitted for simplicity and practicality.

Multi-criteria analyses do come at a cost. In their current form, unlike cost–benefit and cost-effectiveness analyses, multi-criteria models do not explicitly guide resource allocation. No widely accepted rule exists for multi-attribute approaches that match the logical ease and spirit of “invest when the net benefits are positive” (as in cost–benefit analysis) or “approve the project if the cost per unit of health gained falls below some predetermined cutoff” (as in 1X to 3X per capita GDP for cost-effectiveness analysis). A recent task force of the International Society for Pharmaceutical and Outcomes Research (ISPOR) concluded that the best practices to support the use of multi-criteria decision analysis to consider budget constraints is “still unclear, and further research should focus on this topic” [19]. This paper seeks to contribute to that discussion.

## Methods

As a starting point for discussion, resource allocation relates to how basic measurements are done. With a fixed budget, if all one desires is a prioritized rank order of investments, then ordinal scales (placing choices in the desired order) or interval scales (such as Fahrenheit and Celsius temperatures) suffice. Many forms of multi-criteria decision analysis produce interval scales. In this setting, investments are made until the fixed budget is exhausted.

A more refined approach might allocate a fixed investment budget across scalable investments by choosing how large or small each potential project might be to maximize the overall value of the investments. Assigning appropriate budget shares to each potential investment option requires that the multi-attribute model provide a ratio scale, not just an interval scale. Finally, if one wishes to have a clear decision about whether or not to invest—analogous to the outcome of cost–benefit analysis—then benefits and costs must be measured in the same monetary units (e.g., dollars, euros, yuan or rupees). Since multi-criteria models do not automatically convert benefit measures to monetary units, and often only determine ordinal priority ranks, they do not yet provide generalizable resource allocation rules.

In some settings, resource allocation does not enter the picture. For example, an individual health care patient choosing among insured treatment alternatives need not consider resource costs, but rather selects among the options provided by the patient’s health plan. But in most real decisions—such as insurance coverage decisions and health technology assessment—budget constraints invariably enter the picture. In our own recent work focused on systems analysis for prioritizing new vaccine research and development, the issue of choosing investments within a fixed budget—or determining the level of such a budget—was not a design factor [15, 20, 21]. But in some settings, more guidance about resource allocation is needed.

The recent ISPOR task force assessing multi-criteria decision analysis models discussed this challenge, summarizing previous work on the topic and offering three alternatives (none of which they deemed wholly satisfactory), and urged further research on the issue [19]. The approaches they identified from that literature survey included the following:

### Directly include costs

One approach to solving this problem directly includes cost as an attribute in the multi-criteria analysis—lower costs being better—with a user-assigned weight within the model. This has the equivalent effect of asking the decision maker (when establishing the weights) to evaluate willingness to pay for the benefits. But as the ISPOR task force report notes “stakeholders do not have the knowledge to estimate the benefits that would have to be forgone to fund an alternative. Instead, this would require the forgone alternatives to be identified and evaluated using the same” multi-criteria framework.

### Score comparable interventions

The scoring approach seeks to find existing interventions that might be eliminated to free-up funds for the new investment, hence identifying the “opportunity cost” of the new intervention. The scores for the candidates are generated by the multi-criteria model providing a comparative view of their performances. But this approach contains circular logic: how does one know in which programs to disinvest until all programs have been evaluated in the same multi-criteria metric? Those interventions that may seem available for elimination in cost-effectiveness analysis may look very good in a multi-criteria model, and vice versa. So the selection of a group of comparable interventions is an incomplete and defective approach. Rankings of investment priorities can readily shift importantly when other criteria—such as public fear during a disease outbreak—enter the analysis beyond cost-effectiveness [10].

### Modified cost–benefit calculations

This approach omits “cost” in the multi-criteria model, evaluates each of the various options, and then calculates a cost–benefit ratio, similar to an incremental cost-effectiveness ratio. The only difference is that here, the “benefit” metric has multiple dimensions—unlike the unidimensional QALY, DALY, or similar health benefit measure in cost-effectiveness analysis. Multi-attribute models create an index specific to each decision makers’ preferences, and thus such indexes are not comparable with one another. But in this situation, unless all multi-attribute evaluations use the same ratio scale measurement, comparing cost–benefit ratios is impossible. Yet, forcing all measurements into a single multi-attribute framework defeats its very purpose—that is, allowing different stakeholders the ability to specify their own preference functions.

In a similar fashion, one recent analysis recommends calculating the ratio of multi-criteria value scores to cost [22]. This approach then sorts the available choices from the most favorable to the least favorable, and then proceeds until the investment budget is exhausted. Unfortunately, this approach does not provide advice on the proper investment budget size—it is exogenous in their analysis. Nor does it allow for the possibility that at least some of the possible investments are scalable, which would introduce further investment options beyond those originally considered. This rule is akin to calculating QALYs per cost (the inverse of the usual metric) and investing in the most favorable until the budget is exhausted. It provides neither a cutoff rule—as has become common in cost-effectiveness analysis—nor a mechanism for budget setting mechanism; so while it places organizations on the efficient frontier, it does not identify the best part of that frontier. Economists call this technical or “X-efficiency” but it is an incomplete measure of overall efficiency, since it ignores “allocative efficiency” [23].

In real world settings, budgets do not ultimately descend from the top: somebody has to work through and determine the desired level of investment. Thus something further is needed to guide such decisions. To make further headway, we need an approach to guiding either cutoff values or a mechanism for shaping an optimal budget. The two tasks cannot be done independently—one implies the other.

### League tables

One could also construct a league table for multi-attribute models of various interventions to provide guidance, just as early cost-effectiveness analysis used league tables to guide resource allocation before people became comfortable with choosing a cutoff value. Earlier use of league tables for cost-effectiveness contains a strong assumption, namely that previous decisions about health care interventions were made with implicit cost-effectiveness tradeoffs in mind. But if those league tables also included (even informally) the value of other attributes, then they could overstate the willingness to pay for a QALY. This approach is logically equivalent to one of the approaches evaluated by the ISPOR task force and it contains the same defect. One does not know *ex ante* which technologies appropriately belong in the league table and which are simply out of bounds.

### Willingness to pay and accept

As a proxy for resource allocation, one could also simply survey the measures of willingness to pay (WTP) and willingness to accept (WTA), as is common, for example, in environmental policy [24]. However, behavioral scientists cast serious doubt on the validity of such approaches, arguing that the responses may reflect attitudes, but do not represent true willingness to pay [25]. A prominent concern is that of framing, where responses depend on the way the question was posed. The distinction often hinges on WTP (as in “how much would you be willing to pay to avoid unpleasant situation X”) versus WTA (as in “how much would you need to be paid before you would accept unpleasant situation X”). Answers differ greatly in these issues depending on the framing: whether or not the object is currently owned, and is available or not.

A review comparing numerous WTA and WTP studies on the same economic area (e.g., health, environmental) concluded that WTA values regularly exceed WTP values, the gap highest for non-market goods. The authors of this summary concluded that the less the good is like an ordinary market good—that is, it cannot be readily be bought and sold—the higher the ratio [26]. They reported a typical WTA/WTP ratio of 7.2 for analyses carried out in a number of different subject areas (including health, environmental, water resources and others). A more recent review found a WTA/WTP ratio of 5.1 for goods involving health and safety [27]. These results appear to show that WTA studies—such as those involving wage premiums for risky occupations—severely overstate the more desirable measure of willingness to pay. If so, then relying on these WTA measures instead of agreed-upon cost per QALY measures would be inappropriate.

Since “health” is perhaps the quintessential non-market good, one might expect the WTA/WTA ratios for the value of life and life years to possibly be significantly higher than for typical market goods. However, some proponents of the use of labor force studies (wage differentials for risky occupations) to measure value of life argue that the gap is not nearly so large as this literature suggests, if properly interpreted, thus seeking to restore confidence in the large value of life measures found in the health and safety literature [28].

## Results

Our approach requires that any multi-criteria decision model contain a component of health benefits for which there is at least some agreement regarding a proper cutoff for cost-effectiveness analysis. Suppose that the health benefits measure (e.g., QALY or DALY) has a weight *w*, and all other attributes combined have a weight of (*1* − *w*). If the agreed upon cost-effectiveness cutoff is to accept any intervention with cost per QALY (or DALY) ≤ K, then the proper cutoff in the multi-criteria model is K/*w.* K is the more binding of the announced cost-effectiveness threshold or the implicit and more stringent value from a tight budget.

Using QALYs (or DALYs) as a standard of value, we can scale the total willingness to pay for the aggregate benefit by using the fraction of the total benefit attributable to QALYs (or DALYs). Our proposal therefore leverages previous agreement about proper cost-per-QALY threshold into a new threshold for the newly defined portfolio of benefits. The value of QALY serves as the numeraire.

Suppose two decision makers have created their respective multi-attribute models where QALYS account for different percentages of the total value weight. In Table 1, these two decision makers are presumed to agree on the proper cutoff for a cost-effectiveness model at $100,000 per QALY. This generalizes to the situation where they have different initial cutoffs, as shown in Table 2. In both cases (Tables 1 and 2) once we know the decision maker’s cutoffs for cost-effectiveness and their weights assigned to health outcomes in the multi-criteria analysis, then we can infer the proper cutoff for decision making using multi-attribute models.

**Table 1:** Comparison of multi-criteria cut-off points for 2 hypothetical decision makers with same starting point cost-effectiveness cut-offs

	Weight on QALYs	Weight on other attributes	Cost-effectiveness ($/QALYs) cutoff	Multi-criteria cutoff
Decision maker A	0.5	0.5	$100K	$200K
Decision maker B	0.666	0.333	$100K	$150K

**Table 2:** Comparison of multi-criteria cut-off points for 2 hypothetical decision makers with different starting point cost-effectiveness cut-offs

	Weight on QALYs	Weight on other attributes	Cost-effectiveness ($/QALYs) cutoff	Multi-criteria cutoff
Decision maker A	0.5	0.5	$80K	$160K
Decision maker B	0.666	0.333	$100K	$150K

Returning to the affordability conundrum raised earlier, the notion that one should be willing to pay more for an item with greater value seems incontestable. However, if a fixed budget health care system suddenly introduces an expanded multi-criteria measure of value (and logically, a greater willingness to pay for that expanded concept of value) then the budget constraint will likely become *more* binding, and the gap between the stated willingness to pay and the shadow price in the budget can widen.

To see how this works, suppose that a health care system introduced a multi-criteria model with two attributes of value—QALYs gained and the extent to which disease burden of identified disadvantaged populations is equitably reduced. Some interventions that might not meet a population-wide cost-effectiveness criterion might now have higher priority. Tuberculosis prevention or treatment might provide a good example—low priority in a general population but high priority in a disadvantaged population. Using the new measure of value would increase the desire to fund (in our example) the tuberculosis-treatment program, hence further stressing the overall budget for the health care system.

To bring things into alignment logically one of three things must occur: (a) new resources must be added to the budget (b) some previously funded activities must be defunded, or (c) the threshold for accepting interventions must tighten (or some combination of these options). In using this approach in situations with a fixed budget (or until a budget can be adjusted to accommodate new items of value), it is important to use the appropriate threshold, which may well be more stringent than the announced threshold, and which will change even further as the extra value of non-QALY items is introduced into the analysis.

## Discussion

One can ask under what circumstances our simple extrapolation procedure remains valid beyond a simple linear utility model. As a starting point, the extrapolation of the value for QALYs to the entire multi-criteria model remains valid whenever the decision maker’s assumed utility function has constant budget shares (proportions of the total budget spent on a particular good). Cobb–Douglas utility models have this feature—the budget shares are constant over all incomes and prices. A more general set of utility functions—those with constant elasticity of substitution—assures that the method is globally correct while allowing incomes to change, but holding relative prices of QALYs and the other goods constant.

In more generalized utility structures, budget shares vary with changes in income and relative prices of goods. In such cases, the simple proportional extrapolation from the value of a QALY to the value of a more complex multi-criteria bundle will require adopting a specific functional form for the utility structure and then calculating the appropriate extrapolation method.

Multi-criteria models in general are meant to help structure problems for decision makers and to provide general guidance, not to provide precise measures of value. There is always a tradeoff between accuracy and simplicity, and most practitioners of multi-criteria decision analysis generally opt for simplicity when possible. Under what circumstances our extrapolation method remains valid can and should be a topic for further research.

Since much of the literature on choosing a cutoff for an acceptable cost per QALY has focused on its relationship to income, this suggests that our extrapolation method will be reasonably useful even if ignoring differences in relative prices for QALYs and other goods in the multi-criteria bundle.

We also note that even the standard model of cost-effectiveness analysis and the associated “acceptable technology” cutoff rules are not invariant to changes in economic conditions. The current debate about how to incorporate “affordability” into cost-effectiveness analysis highlights this issue. If a new technology emerges that has widespread use yet its cost-effectiveness ratio is “acceptable” by current norms, the situation can easily arise where the technology is both acceptable and unaffordable (with a fixed budget). If the underlying economic conditions change markedly (income, prices, or technological opportunities), then the original behavioral rules that emerge (e.g., a cost per QALY rule) must be revised. This is true both in a pure QALY-based model and in our more general model that incorporates both QALYs and other goods.

## Conclusion

The basic idea of our approach is straightforward: If one knows the value of part of a package of valuable items, and one knows the proportion of overall value of the package attributable to that particular part of the package, then one can readily deduce the overall value of the package. In the realm of health, the most likely components to serve this purpose appear to be QALYs or DALYs. The benefit of using existing cutoff measures such as cost per QALYs is simply that a considerable literature exists on determination of those values. We note—referring to the obvious—that the difficulty in reaching agreement about the proper cost per QALY threshold suggests that reaching consensus about a cutoff for multi-attribute decision models may be even more difficult.
